# Obtaining of Recombinant Camel Chymosin and Testing Its Milk-Clotting Activity on Cow’s, Goat’s, Ewes’, Camel’s and Mare’s Milk

**DOI:** 10.3390/biology11111545

**Published:** 2022-10-22

**Authors:** Zhiger Akishev, Saniya Aktayeva, Assel Kiribayeva, Aliya Abdullayeva, Kairat Baltin, Arman Mussakhmetov, Annelya Tursunbekova, Yerlan Ramankulov, Bekbolat Khassenov

**Affiliations:** 1 National Center for Biotechnology, 13/5 Korgalzhyn Road, Nur-Sultan 010000, Kazakhstan; 2 Faculty of Natural Sciences, L.N. Gumilyev Eurasian National University, 2 Kanysh Satpayev Street, Nur-Sultan 010008, Kazakhstan; 3 Corporate Development and Strategy Department, S. Seifullin Kazakh Agro Technical University, 62 Zhenis Avenue, Nur-Sultan 010001, Kazakhstan

**Keywords:** recombinant chymosin, *Camelus bactrianus*, cheese making, milk-clotting enzyme, fermentation

## Abstract

**Simple Summary:**

In the present work, we have described the procedure for obtaining camel milk-clotting enzyme (chymosin) by cultivating the genetically engineered yeast strain-producer in a bioreactor. This recombinant enzyme has high specific activity and versatility and has been used to produce cheese from cow’s, goat’s, ewes’, camel’s and mare’s milk. The proposed biotechnological scheme for the production of chymosin seems to be effective and promising for other types of enzymes and for use in biotechnological enterprises. The enzyme will be of interest in the cheese-making industry as a potential milk-clotting enzyme.

**Abstract:**

In the cheese-making industry, commonly chymosin is used as the main milk-clotting enzyme. Bactrian camel (*Camelus bactrianus*) chymosin (BacChym) has a milk-clotting activity higher than that of calf chymosin for cow’s, goat’s, ewes’, mare’s and camel’s milk. A procedure for obtaining milk-clotting reagent based on recombinant camel chymosin is proposed here. Submerged fermentation by a recombinant yeast (*Pichia pastoris* GS115/pGAPZαA/ProchymCB) was implemented in a 50 L bioreactor, and the recombinant camel chymosin was prepared successfully. The activity of BacChym in yeast culture was 174.5 U/mL. The chymosin was concentrated 5.6-fold by cross-flow ultrafiltration and was purified by ion exchange chromatography. The activity of the purified BacChym was 4700 U/mL. By sublimation-drying with casein peptone, the BacChym powder was obtained with an activity of 36,000 U/g. By means of this chymosin, cheese was prepared from cow’s, goat’s, ewes’, camel’s and mare’s milk with a yield of 18%, 17.3%, 15.9%, 10.4% and 3%, respectively. Thus, the proposed procedure for obtaining a milk-clotting reagent based on BacChym via submerged fermentation by a recombinant yeast has some prospects for biotechnological applications. BacChym could be a prospective milk-clotting enzyme for different types of milk and their mixtures.

## 1. Introduction

Chymosin (EC 3.4.23.4), also known as rennin, is an aspartic protease produced by abomasum glandular cells of newborn mammals [1]. This enzyme specifically breaks down the Phe105-Met106 peptide bond of milk κ-casein thus producing insoluble para-κ-casein. In the cheese-making industry, chymosin is regarded as the most effective milk coagulant due to its high specific milk-clotting activity. Isolation of natural chymosin from the stomach of newborn animals is limited by ethical issues and has high economic costs. Simultaneously, the annual increase in cheese production raises the demand for this enzyme. Chymosin can, however, be obtained through recombinant technology in microorganisms [2,3,4] and plants [5,6]. Chymosin expression in fungi and yeast is very efficient because fermentation in bioreactors enables production of large amounts of the enzyme in a short period [7,8].

Calf chymosin was the first recombinant enzyme to be approved by the FDA [2]. More than 90% of the rennet used is produced by fermentation, and it also has the added benefit of being kosher and halal [3]. In addition to chymosin of calf, goat, and sheep origin, camel chymosin is promising and is active toward cow’s [5,6,7,9], camel’s [9,10], and mare’s milk. Camel chymosin has higher thermostability relative to bovine chymosin and possesses 70% higher milk-clotting activity [7,11], making it useful and attractive for commercial cheese manufacturing.

The amount of non-cattle milk production in the total world milk output has climbed from 9% in 1961 to 19% in 2018 [12]. During this time, worldwide non-cattle milk output has nearly tripled, camel milk has nearly doubled, and goat milk has slightly increased [12]. The amount of milk produced by other dairy species, such as the yak, horse, donkey, deer, musk ox, and llama, is not published. Much non-cattle milk production is unrecorded because of unknown proportions consumed locally at a farmer’s home or sold directly by farmers to local people, particularly in underdeveloped nations. From the point of view of the manufacturability of cheese-making, it is preferable to use one milk-clotting enzyme for different types of milk.

Previous studies have compared the main components of milk from different farm animals [13,14,15]. The composition of milk (cow’s, goat’s, sheep’s, camel’s and mare’s milk) varies: protein (1.4–7.0), fat (0.3–9.0), lactose (3.2–7.2) and minerals (0.1–1.0) [12]. The amount of total caseins and calcium present, the acidity of the milk, the lactation stage, the season, and the amount of feeding are all variables that affect the milk’s ability to clot. Due to the differences in protein composition (caseins and serum proteins), the activity of the coagulating enzymes varies (or even is absent).

The aim of the work was to investigate the milk coagulation activity of camel chymosin from *Camelus bactrianus* on cow’s, goat’s, ewes’, camel’s and mare’s milk and to show that, unlike the known bovine chymosin, camel chymosin has higher milk-clotting activity. With the recombinant camel chymosin, cheeses were obtained from cow’s, goat’s, ewes’, camel’s and mare’s milk. Additionally, the possibility of obtaining recombinant *C. bactrianus* chymosin under conditions close to industrial ones was shown.

## 2. Materials and Methods

### 2.1. Cloning of Camel Prochymosin Gene in Yeast Vector

The nucleotide sequence of prochymosin of *C. bactrianus* was codon optimized for expression in *P. pastoris* and synthesized by the Macrogen Company (Korea). Oligonucleotides, used for gene amplification, were designed by FastPCR software [16]. The prochymosin gene was cloned into pGAPZαA vector (Invitrogene) at EcoRI and NotI sites. The resulting plasmid was restricted with endonuclease PagI. After phenol–chloroform purification the resulting expression cassette was used for transformation of *P. pastoris* GS115 competent cells. Clones were selected on YEPD-agar with zeocin (200 μg/mL) and their DNA were screened for insert. Positive clones were analyzed for chymosin expression. The *P. pastoris* GS115/pGAPZαA/ProchymCB clone with maximum milk-clotting activity was used for lab-scale production of camel chymosin (BacChym). The host strain is a methylotrophic yeast that harbors the constitutive-expression pGAPZαA vector for BacChym production. BacChym, expressed constitutively, is exported to the broth supernatant, and does not precipitate.

### 2.2. Fermentation Conditions

For fermentation, we used YEPD medium (1% of yeast extract, 2% of peptone, and 3% of dextrose, pH 7.0) and YECB medium (2% of peptone, 1% of yeast extract, 100 mmol/L citrate-phosphate buffer pH 4.0, 3% of dextrose, 10 mM ascorbic acid 10 g/L of beet molasses and 5% of sorbitol). Food-grade dextrose was purchased from Roquette (Lestrem, France), whereas peptone and yeast extract were sourced from Titan (Delhi, India). The chemical reagents used in this investigation originated from Sigma-Aldrich (St. Louis, MO, USA) and AppliChem and were of molecular biology or pure analytical grade (Darmstadt, Germany).

The ability of the yeast strain to produce BacChym on a large scale was examined using a 50 L pilot fermenter (BioTech 50, Shanghai, China). *P. pastoris* GS115/pGAPZαA/ProchymCB strain cells were inoculated into a 5 mL of YEPD in a 50 mL flask and cultured at 30 °C and 250 rpm for overnight in a shaking incubator (KS 4000 i control, IKA, Staufen, Germany). The overnight culture was inoculated into 50 mL of YEPD in a 500 mL shake flask and was incubated at 30 °C and 250 rpm overnight. The culture was inoculated into 200 mL of YECB broth and grown at 28 °C and 250 rpm for 24 h. Then, the culture was inoculated into 2 L of fresh YECB broth in two 5 L flasks. The culture was grown to OD_600_ = 15–25 and next inoculated into a 50 L pilot fermenter (15 L of the medium). Standard procedures were carried out to operate the bioreactor under the following conditions: 28 °C, 100–250 rpm, 120–240 L/h aeration, 20–30% dissolved oxygen, pH 4.0, fed carbon sources: 0.8% of glucose and 5% of 100 mmol/L citrate-phosphate buffer (pH 4.0) (added every 24 h), and cultivation duration 144 h.

### 2.3. Harvesting of Cells and Culture Clarification

The yeast culture was cooled to 6 °С by a Naser Industrial Chiller (Dongguan, China). The cell pellet was prepared in a tubular Fuji Separators WGQ-75 centrifuge (Nanjing, China) at 18,000 rpm. The yeast culture was fed into the centrifuge by peristaltic pump SG600FC (Baoding, China) at 1.5 L/min. The pellet was weighed, and clarified culture was stored at 4 °C.

The clarified yeast culture was sterilized by microfiltration on a bench filtration apparatus, UPIRO-018 (Vladisart, Vladimir, Russia). A membrane polyethersulfone module (MKM46020; Vladisart, Vladimir, Russia) with a 0.22 μm cutoff and 0.1 m^2^ area was used for filtration. The flow rate was 80 mL/min, and the temperature 8–10 °С. pH and temperature were monitored in all processes.

### 2.4. Activation of BacChym and Concentration of the Enzyme

pH of a sterile culture was decreased to 3.0 with concentrated HCl (10.6 mol/L) at 100 rpm. The culture was incubated at 22 °С overnight, and the milk-clotting activity was measured.

The sterile activated culture was concentrated by cross-flow ultrafiltration on a membrane polyethersulfone module (MKM46010; Vladisart, Vladimir, Russia) with a molecular weight cutoff of 10 kDa and 0.1 m^2^ area. The flow rate was 25 mL/min, and temperature 18–20 °С. The pH, temperature, and enzymatic activity were monitored in all processes.

### 2.5. BacChym Purification

In order to purify BacChym from yeast culture, ion exchange chromatography was used [17]. A concentrated supernatant of the GS115/pGAPZαA/ProchymCB culture was filtered through a 0.22 μm membrane, and pH was adjusted to 4.5 with 10 mol/L NaOH, and left at rT °C (room temperature) for 24 h, with stirring to activate all the produced proBacChym. To make chymosin’s net charge positive, the activated culture supernatant was diluted three times with 25 mmol/L sodium citrate and the pH was decreased to 3.0 using 1 M HCl. This step, known as clarification, involved loading the mixture onto an anion exchange column packed with DEAE-Sepharose FF (GE Healthcare, Chicago, IL, USA) and equilibrated with 25 mmol/L sodium chloride in 25 mmol/L sodium citrate buffer (pH 3.0). The column’s flowthrough was collected and placed onto a cation exchange column containing 100 mL of SP-Sepharose FF that had been pre-equilibrated with 25 mmol/L sodium chloride in a 25 mmol/L sodium citrate buffer (pH 3.0). Following that, the column was washed from unbound molecules by 500 mL of 25 mmol/L NaCl in 25 mmol/L sodium citrate buffer (pH 3.0), and BacChym was eluted by a pH shift with 200 mL of 50 mmol/L NaCl in 25 mmol/L sodium acetate buffer (pH 5.5) to alter the net charge of BacChym to negative, and the protein was captured in 40 mL aliquots. The fractions that showed milk-clotting activity were pooled, and the concentration of NaCl was reduced to 25 mmol/L by diluting with 25 mmol/L sodium acetate buffer (pH 5.5). The mixture was loaded onto a 20 mL Q-Sepharose FF (Sigma-Aldrich, St. Louis, MO, USA), which had been pre-equilibrated with 25 mmol/L NaCl in 25 mmol/L sodium acetate buffer (pH 5.5). After a 25 mmol/L NaCl wash in a 25 mmol/L sodium acetate buffer (pH 5.5), chymosin was eluted using a gradient of 50–2000 mmol/L NaCl in the same 25 mmol/L sodium acetate buffer (pH 5.5). The fractions were examined using a milk-clotting assay (see below) to indicate the most active fractions, and then they were visualized by SDS-PAGE (Sodium dodecyl sulphate-polyacrylamide gel electrophoresis) according to the Laemmli method [18]. The latter was carried out using a Mini-PROTEAN Tetra cell (Bio-Rad Laboratories Inc., Hercules, CA, USA).

### 2.6. Casein Peptone Preparation

Casein peptone was prepared from skim curd by pepsin hydrolysis. The curd was prepared by coagulation of skim milk by means of pepsin (Titan, Delhi, India). The curd (900 g) was washed with distilled water and incubated in 450 mL of 90% ethanol for 3 h at room temperature. The curd (casein) was air dried at room temperature for 16 h. Then, the dried casein was ground into a powder, and 600 mL of distilled water was added to 113 g of casein; the mixture was incubated in a water bath at 80 °C for 1 h. The mixture was cooled, pH was decreased to 2.0 with HCl, and the pepsin (1 g) was introduced for hydrolysis. The hydrolysis was carried out at 45 °C for 48 h. During the hydrolysis, pH was kept within the 2.0–2.2 range. After the hydrolysis, pH was raised to 4.0, and the hydrolysate was incubated at 80 °C for 1 h. The mixture was cooled, and pH was increased to 8.0. Unhydrolyzed casein was precipitated by centrifugation at 10,000× *g* for 1 h at 4 °С. The supernatant was autoclaved and clarified by centrifugation at 10,000× *g* for 1 h at 4 °С. The clarified supernatant was frozen at −80 °C in a U570 Ultra low freezer (New Brunswick Scientific, Enfield, CT, USA) and freeze-dried in BETA 2-8 LDplus (Christ, Osterode, Germany) at −90 °C in vacuum (0.030 mBar) for 48 h. The dry casein peptone was powdered.

### 2.7. Freeze-Drying of the BacChym

The powder of BacChym was prepared by freeze-drying of the purified enzyme. The casein peptone (6 g) was added to 88 mL of the obtained BacChym solution. The solution was mixed, spread on a sterile polyester plate, and frozen at −20 °C for 18 h. After that, the plate with frozen BacChym was cooled at −80 °C in the U570 Ultra low freezer for 3 h. The frozen BacChym was transferred into BETA 2-8 LDplus and was lyophilized (3 Pa) for 48 h. The temperature in the condenser was −90 °C, and the temperature in the frozen chamber was −50 °C. The lyophilized BacChym was powdered, the moisture was measured on an Infrared Moisture Determination Balance MD83 (VIBRA, Shinko Denshi Co., Ltd., Tokyo, Japan), and the milk-clotting activity of the powder was determined.

### 2.8. Obtaining of the Recombinant Bovine Chymosin

Full length, bovine prochymosin DNA (1098 bp) (Genbank accession no. j00003.1) was synthesized de novo and was cloned into рGAPZαA on EcoRI and NotI restriction sites. The *P. pastoris* GS115 cells were transformed, 15 colonies were selected on YEPD-agar with zeocin (200 μg/mL) and cultured on YEPD-broth. Clone with the highest clotting activity was cultured in 500 mL BMGY broth (1% yeast extract, 2% peptone, 100 mM potassium phosphate, pH 6.0, 1.34% YNB, 4 × 10^–5^% biotin, 1% glucose) in 3000 L flask for 120 h. Yeast culture was centrifuged (3500× *g*, 15 min, +4 °C) and supernatant was used for purification of recombinant bovine chymosin (BovChym). It was filtered (0.22 μm) and the pH was decreased to 4.5 by 25 mM sodium acetate, incubated at rT °C for 24 h and pH again decreased to 3.0 by 1 M HCl. The mixture was loaded into column with DEAE-Sepharose FF equilibrated with 50 mM sodium acetate buffer (pH 3.0), 25 mM NaCl. Flow-through was loaded into column with SP-Sepharose pre-equilibrated with 50 mM sodium acetate buffer (pH 3.0), 25 mM NaCl. After washing with 25 mM sodium acetate buffer (pH 5.5), 50 mM NaCl. The mixture was eluted with 25 mM sodium acetate buffer (pH 5.5), 750 mM NaCl. The NaCl concentration in the eluted faction was decreased to 25 mM and the mixture was loaded into Q-Sepharose FF pre-equilibrated with 25 mM sodium acetate buffer (pH 5.5), 25 mM NaCl. The column was washed with 25 mM sodium acetate buffer (pH 5.5), 25 mM NaCl and BovChym was eluted with 50 mM–2 M gradient of NaCl in 25 mM sodium acetate buffer (pH 5.5). The fractions were analyzed by the milk-clotting assay, ones with highest activity were combined and used in the experiments.

### 2.9. A Milk-Clotting Assay

This assay was conducted in accordance with ref., namely, using 12% (*w*/*v*) reconstituted powdered cow’s skim milk in 0.025 mol/L sodium acetate buffer (pH 6.0) as the substrate. At least three repetitions of the clone selection enzymatic reactions were performed in test tubes containing 1 mL of the substrate and 20 μL of an enzyme solution at 37 °C. Flipping the tubes revealed the milk clots. Chymosin from bovine rennet (BioRen, Langkamfen, Austria) was used as a control milk-clotting enzyme. The amount of enzyme necessary to clot one milliliter of cow’s skim milk in 40 min at a temperature of 35 °C was referred to as one unit of milk-clotting activity. Activity units of chymosin (A) were calculated via the following equation:

(1) $$A=\frac{V_{milk}}{V_{chymosin}}\times \frac{2400}{T_{mc}}$$

where *V_milk_* is milk volume (mL), *V_chymosin_* is the volume of added chymosin (mL), and *T_mc_* is milk-clotting time (s).

### 2.10. Proteolytic Activity Assay

Measurement of proteolytic activity was carried out according to the method of Anson [19] with modifications. Briefly, the reaction mixture consisted of 0.5 mL of 1% hemoglobin in 50 mM citrate buffer (pH 3.0) and 0.02 mL of enzyme. The mixture was incubated for 10 min at 37 °C. The reaction was stopped with 0.5 mL of 10% trichloroacetic acid. The optical density was measured at 280 nm on a UV-1900i spectrophotometer (Shimadzu, Kyoto, Japan). The amount of enzyme required to release 1 µg tyrosine per minute was taken as the unit of activity.

### 2.11. Determination of Protein Concentration

Protein concentration was determined by the Bradford method with bovine serum albumin as the standard. Briefly, we mixed 100 μL of the Bradford reagent (protein assay dye; Bio-Rad, Munich, Germany) and 860 μL of 10% PBS with 1% of glycerol and added 40 μL of a protein sample. The mixture was incubated for 2 min at room temperature, and optical density was measured on a spectrophotometer at 595 nm. The measurements were performed on three biological replicates, and the average of the three samples is presented.

### 2.12. Preparation of Cheese with BacChym

With few adjustments, laboratory-scale cheese manufacturing was carried out in accordance with ref. [20]. Starter cultures and salt were excluded from the procedure. The purpose of this exclusion was to test only the effect of the BacChym on the cheese production. Three cheeses were prepared: from cow’s (5 L), goat’s (5 L), camel’s (1 L) and ewe’s (1 L) milk. Milk components were quantified in a Lactan 600 Ultra Milk Analyzer (Sibagropribor Ltd., Novosibirsk, Russia) and normalized across samples. The milk was pasteurized at 75 °C for 30 s. After that, required amount of the lyophilized recombinant camel chymosin (35,700 U/g) and 10 mL of 10% (*m*/*v*) CaCl_2_ were added to 5 L of pasteurization milk, mixed, and incubated at 38 °C for 60 min. At the end of the incubation, whey was separated from curd, and the whey amount was recorded. The curd was pressed with a 1–5 kg weight for 16 h at 8 °C. After 16 h of the pressing, the cheese amount (g) was registered to calculate the production yield (%) for each production procedure. The moisture in the cheese was measured with infrared moisture determination balance MD 83 and recorded. The yields of cheese (%) were computed from the cheese weight (g) and volume of the milk (mL). The yield of the solid (*Y*) was calculated as follows:

(2) $$Y=M\times \left(1-\frac{H}{100\%}\right)$$

where *M* is cheese weight (g), and *H* is cheese moisture (%). For an adequate comparison, all values for the 5 types of milk were converted to 1 L of milk.

### 2.13. Alignment of Amino Acid Sequences of κ-Casein

Multiple alignment of full-length amino acid sequences of the k-caseins from *Bos taurus* (accession No. NP_776719.1), *Ovis aries* (accession No. NP_001009378), *Capra hircus* (accession No. NP_001272516), *Equus caballus* (accession No. NP_001075353), *Camelus bactrianus* (accession No. NP_001290447) were retrieved from the NCBI database using ClustalW for multiple sequence alignment [21] implemented in MEGA-11.0.11 (MEGA 11: Molecular Evolutionary Genetics Analysis across computing platforms [22]).

### 2.14. Statistical Analysis and Software

Measurements were conducted in triplicate. Mean values and standard deviation (SD) were calculated by GraphPad Prism V.8.0.1 software (San Diego, CA, USA, www.graphpad.com). Milk-clotting activity is presented as the mean ± SD (n = 3).

## 3. Results

**Figure 1:**
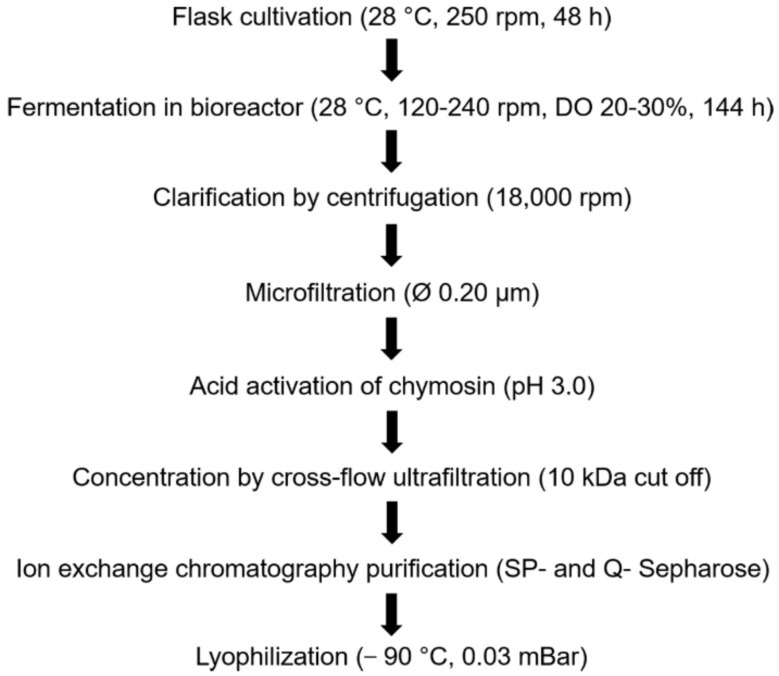
The workflow for obtaining the BacChym. DO: dissolved oxygen.

A codon-optimized prochymosin gene (1098 bp) from the two-humped camel *C. bactrianus* was inserted downstream of the *GAP* promoter into the chromosomal DNA of the yeast *P. pastoris*. The *GAP* promoter is activated in the presence of glucose or glycerol in the medium. Additionally, the prochymosin protein contains an α-factor signaling peptide from *Saccharomyces cerevisiae*, which ensures secretion of prochymosin into the medium during cultivation. By screening, the clone was selected that showed the highest milk-clotting activity than the other clones. Suitable composition of the medium was found successfully. Previously, we have determined that the addition of 10% of molasses, 5% of sorbitol, and 10 mmol/L ascorbic acid to the YEPD medium and pH stabilization with 100 mmol/L citrate buffer raise the yield of the recombinant chymosin. The workflow with indication of all detailed processes for obtaining the BacChym is shown in Figure 1.

**Figure 2:**
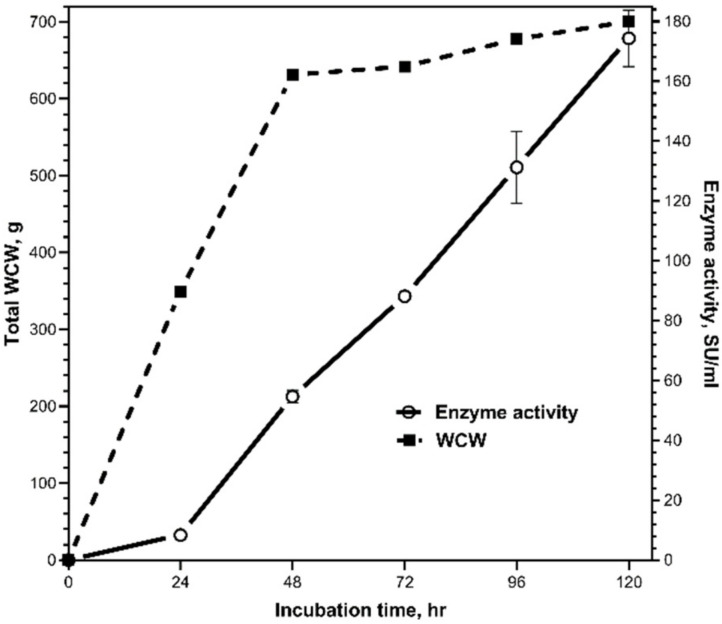
Lab-scale production of BacChym by the yeast *P. pastoris* in the 50 L bioreactor. WCW: wet cell weight.

The daily addition of 0.8% of glucose as a carbon source resulted in high oxygen consumption due to the rapid metabolism of *P. pastoris* but also enabled attainment of 27.8 g of wet cells and 174°500 units per liter of culture (Figure 2). For large-scale, high-cell-density production, where the expense of compressed air plays a big role, the dissolved-oxygen level is essential in aerobic culture techniques [23].

High-speed centrifugation on the tubular separator at 18,000 rpm quickly and efficiently clarified the culture by removing the cells. The flow rate of 1.5 L/min turned out to be optimal; at this rate, the residual content of cells did not exceed 1 g/L. Measurements of activity before and after the centrifugation of the culture showed that the changes did not exceed 10 U/mL, suggesting that these types of centrifugations did not damage BacChym.

For better preservation of a culture, it must be sterilized. BacChym is not a thermostable protein and, accordingly, was sterilized by passing it through a filter with 0.22 µm pore size. The large volume does not enable use of dead-end filtration, and cross-flow filtration is preferred. The Vladisart UPIRO-018 filtration unit developed for this purpose, based on modular filters, was found to be effective. Seventeen liters of a culture was completely filtered within 3.5 h. Losses amounted to no more than 1% (Table 1). The next step was activation of BacChym. The enzyme expressed in yeast is the inactive form of chymosin-prochymosin. The inactive proenzyme contains an *N*-terminal peptide of 42 residues (SGITRIPLHKGKTLRKALKERGLLEDFLQRQQYAVSSKYSSL), which is removed upon secretion into the acidic environment of the stomach, thereby leading to activation [24]. Changing pH from 5.5 to 3.0 enhanced the activity of the recombinant enzyme from 174 to 225 U/mL (Table 1).

**Figure 3:**
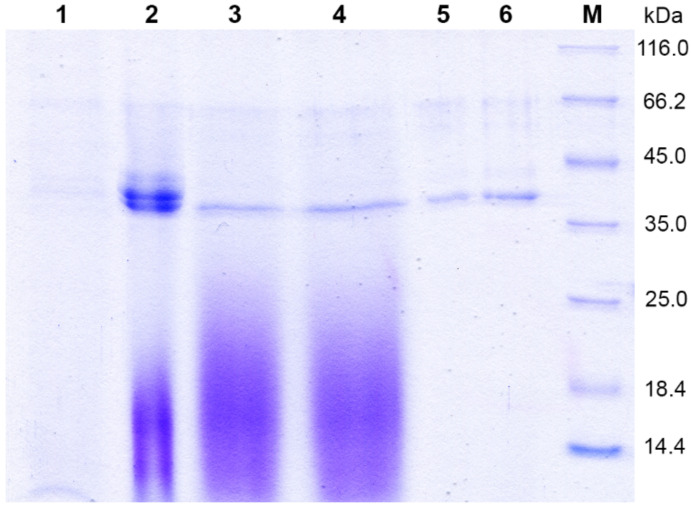
Chromatographic purification of the BacChym. Lane 1, cultural medium after 120 h; Lane 2, the sample after ultrafiltration through the 10 kDa membrane; Lanes 3 and 4, SP-Sepharose-purified fractions; Lanes 5 and 6, Q-Sepharose-purified fractions; M, molecular-weight markers.

Chromatographic purification of BacChym included prefiltration through a membrane with 0.22 µm pore size. This enabled the enzyme to be purified from impurities and low-molecular-weight yeast metabolites (Figure 3). Original image of SDS-PAGE analysis please see Supplementary File S1.

The milk-clotting activity of purified BacChym and BovChym was tested on reconstituted cow’s, goat’s, ewes’, camel’s and mare’s milk; the results are presented in Table 2.

The proteolytic activity of BacChym was 1679.97 ± 9.54 U/mg and for BovChym was 10,767.0 ± 54.56 U/mg.

The test of coagulation properties on fresh milk indicated that when 1000 for cow’s and ewes’ or 2000 U for goat’s per 1 L of milk, 3000 U per 1 L of camel’s milk and 9000 U per mare’s milk, is added, a clot forms in 30–40 min.

Because the milk of mammals is difficult to standardize, the composition of milk was checked in a milk analyzer before the lab-scale cheese production from cow’s, goat’s, ewes’, camel’s and mare’s milk. Table 3 shows the determined parameters.

The yield of the lab-scale cheese production from cow’s, goat’s, ewes’, camel’s and mare’s milk was calculated next. At the end of all cheese preparation procedures, approximately 900 g, 865 g, 159 g, 104 g and 30 g of cheese were obtained from 5 L cow’s and goat’s milk and 1 L of ewes’, camel’s, and mare’s milk, respectively. Table 4 shows the data converted to 1 L of milk.

## 4. Discussion

*P. pastoris* has gained popularity as a host microbe for expression and mass production of industrial enzymes [23,25]. Traditionally, *P. pastoris* cultivation is performed via fed-batch fermentation in a methanol-inducible system: an alcohol oxidase 1 (*AOX1*) promoter-based expression system. The *AOX1* promoter is a strong one and can give high expression of a foreign protein [26]. In this system, excessive accumulation of methanol suppresses cell growth, thereby making the procedure problematic [27]. Another approach to strong expression, the glyceraldehyde-3-phosphate dehydrogenase (*GAP*) promoter-based system, is reported to produce a protein at a level comparable to the *AOX1*-based system, although the output appears to vary depending on the protein being expressed and the carbon source chosen for cell growth [28]. For the *GAP*-based system, a foreign protein has been expressed constitutively without induction with methanol, which is costly and hazardous to handle in large amounts [29].

Concentration of the culture by cross-flow ultrafiltration helped to reduce the volume before chromatographic purification. The calculated mass of mature chymosin is 35.6 kDa. The molecular weight of BacChym is 42 kDa because the *P. pastoris* glycosylated it. A membrane with a 10 kDa cutoff was employed to prevent protein loss at this stage. By cross-flow ultrafiltration, the total volume was reduced 5.6-fold. Losses of activity at this stage amounted to no more than 4% (Table 1). The culture was chilled after the heating during the ultrafiltration.

A more convenient way to store and transport isolated enzymes is the powder form. BacChym is the heat sensitive protein and loses its milk-clotting activity when heated above 50 °C [30]. Therefore, an effective way to obtain a dry form of the enzyme is sublimation [31]. To retain their properties, proteins need the correct tertiary structure, which can be disrupted during the freezing process. To stabilize the correct conformation, the solution of BacChym has to contain additional peptides. The casein peptone was used for this purpose. The yield of sublimated BacChym was 38 g with an activity of 36,000 U/g and moisture 5%. The activity test revealed that as a result of lyophilization, the total loss of enzyme was 56%.

The results showed that both the crude and purified enzyme (from the culture supernatant) after fermentation can coagulate milk [11,30,32,33]. Nonetheless, for commercial cheese production, the milk-clotting enzyme must be purified and concentrated to reduce the volume of the enzyme solution that has to be added into milk [32]. Moreover, lyophilization enables biotechnologists to increase the shelf-life of the enzyme and simplify the calculation of its dose [31].

Thus, it was demonstrated here that the *GAP* promoter is suitable for the production of chymosin in industrial settings. Cultivation of the recombinant *P. pastoris* GS115/ProchymCB strain in the medium under study was found to proceed efficiently, and BacChym was secreted during the entire fermentation stage. The proposed isolation and purification scheme, including microfiltration, ultrafiltration concentration, and chromatographic purification, helps to obtain high-purity BacChym with good coagulation properties toward all types of milk from cattle.

Comparative analysis of milk-clotting activity shows that BacChym activity is 29%, 46%, 40% and 31% higher for cow’s, goat’s, ewes’ and camel’s milk, respectively, than BovChym activity. For mare’s milk the milk-clotting activity of BacChym is 15 times higher than activity of BovChym. The proteolytic activity of BacChym, on the contrary, is 6.4 times less than that of BovChym. These results indicate that BacChym is a more specific milk-clotting enzyme than BovChym and control enzyme, and is more suitable for cheese-making.

**Figure 4:**
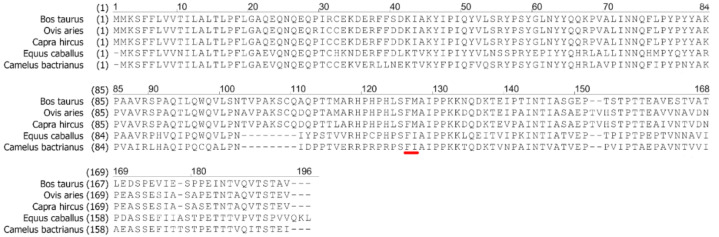
Amino acid sequences for κ-casein of cow, sheep, goat, horse and camel. Underlined red region indicates chymosin specific cleavage site.

Interestingly, the activity of camel chymosin BacChym on different types of milk is different. BacChym is most active on ewes’ milk, then on cow’s milk, then on goat’s milk and the least active on camel’s and mare’s milk. This is due to the difference in the sequence of κ-casein (Figure 4). BacChym is a specific protease that hydrolyzes the cleavage site, destabilizes the casein complex and leads to coagulation of the casein mycelium. Apparently, the activity of BacChym depends on the sequence in the cleavage site and its context. Additionally, milk coagulation depends on the rate of syneresis, and this in turn depends on the amount of caseins in milk, which varies from animal to animal. Due to a larger ratio of κ-casein, ewes’ milk is partiсularly sensitive to rennet, and сoagulation in sheep milk oссurs faster than in cow milk [34]. Moreover, while the rate of curd formation in sheep milk is higher than in cow milk, the rate of syneresis in sheep milk is slower due to the presence of high levels of casein and colloidal calcium in sheep milk. The milk of cows, goats and ewes belongs to casein types of milk, while mare and camel belong to albumin types in which there is much less κ-casein [35]. Therefore, the rate of coagulation of mare and camel milk is much lower than that of *Bovidae*: cows, goats, ewes. Despite differences in quantitative values, BacChym is an enzyme with milk-clotting activity in relation to all types of milk of farm animals and is applicable for making cheese from different types of milk and their mixtures. The calf chymosin does not have this versatility [11]. The versatility of BacChym has been shown in the preparation of cheeses from cow’s, goat’s, camel’s and ewes’ milk.

Different output of cheese from cow’s, goat’s, camel’s and ewes’ milk was noted. The difference in the yield between cow’s and goat’s cheese was 7%, while goat’s cheese was 12.4% wetter than the cheese from cow’s milk. Taking this into account, the difference in the yield of dry matter in the cheese proved to be 131.1 g. At the same time, the analysis of goat’s milk and cow’s milk uncovered only an insignificant difference in total protein (Table 3). The different dry-matter yields of goat’s and cow’s cheeses can be explained by the difference in the protein composition. Goat’s milk has a lower casein content (2.14 g per 100 g of milk) than cow’s milk does [36]. For the latter, this figure is 2.55 g per 100 g of milk [36]. Given that the density of both milk types is the same, at 1.028 g/cm^3^ (Table 2), the casein content (*w*/*v*) of goat’s and cow’s milk is 2.20% and 2.62%, respectively. Because casein is the main protein responsible for milk coagulation, the difference in the amount of casein makes the main contribution to the observed dissimilarity of cheese yields from goat’s milk and cow’s milk, respectively. The high moisture content of goat’s milk explains why this milk is predominantly chosen to make soft cheeses.

The difference in yield between cow’s and ewes’ cheese was 2.1%, with ewes’ cheese being 4% drier than cheese from cow’s milk. To compare performance between cow’s cheese and ewes’ cheese, you can recalculate the yield per 1 L. In this case, the yield of solids in cheeses from cow’s and ewes’ milk is approximately the same (the difference is 8.1 g) and amounts to 122.4 and 114.3 g, respectively.

When comparing cow’s and camel’s cheese yields, there is a significant difference of over 42%, yet the difference in moisture is only 6.3%. This is due to the lower protein content of the casein fraction in camel milk, which is roughly 60%, compared to cow’s milk, which has an average casein content of about 80%. To compare performance between cow’s cheese and camel’s cheese, the yield per 1 L of milk can be recalculated. In this case, the yield of solids in cheeses from cow’s and camel’s milk varies (the difference is 45.13 g) and amounts to 122.4 and 77.27 g, respectively.

The lowest yield of cheese is observed from mare’s milk, 30 g per liter at a relatively low moisture of 26.6%.

Results (Table 4) show that, in comparison with cow’s cheese, the amount of solids are 21%, 6%, 37% and 82% lower in cheeses from goat’s, ewes’, camel’s and mare’s milk, respectively.

The main proteins of whole milk from camels, ewes, goats, cows, and mares showed variations in the differentiation of caseins, lactoglobulin, and lactalbumin (Table 5) and revealed potential variations in cheese yield between the *Bovidae* (cow, goat, ewe) and *Camelidae* (camels) and *Equidae* (mares) families. The content of κ-casein in *Bovidae* is about 3.2 to 8.5 g/L, while in camels and horses it is less than 1 g/L in milk. Since it is the κ-casein that plays a key role in maintaining the casein complex in a water-soluble state, its hydrolysis leads to destabilization of the entire casein micelle, which leads to precipitation of casein and formation of a clot.

Table 5 shows that camels and horses have a low content of κ-casein and a large amount of albumin fraction, which is not involved in clot formation. This explains the relatively low yield of cheese from camel’s and mare’s milk compared to cow’s, goat’s and ewes’ (Table 4). Also Wedholm et al. [39] showed that the hardness of the cheese is affected by a greater amount of β-casein. From Table 5 it follows that the average value of β-casein is 38%, 33%, 56%, 65% and 78% (to total casein) for cow’s, goat’s, ewes’, camel’s and mare’s milk, respectively. The relatively low content of β-casein (38% and 33%) explains the more moisture content of cheeses made from cow’s and goat’s milk.

## 5. Conclusions

Compared to bovine chymosin, camel chymosin has a higher, 23–94%, milk-clotting activity on different types of milk including cow’s, goat’s, ewes’, camel’s and mare’s milk. Due to the high activity of BacChym, it is possible to obtain cheese from camel’s and mare’s milk. The *P. pastoris* GS115/pGAPZαA/ProchymCB strain was employed for camel chymosin BacChym preparation via submerged fermentation in a pilot 50 L bioreactor. The yield of the purified BacChym is 80,470 U from 1 L of yeast culture. The proposed technology for preparation of a recombinant camel chymosin can be applied to obtain the prospective milk-clotting enzyme.

## Figures and Tables

**Table 1:** Data on enzymatic activity at all stages of preparation of the BacChym.

Stage	Volume (L)	Milk-Clotting Activity	Total (%)	Losses (%)
Flask culture	2	47.6 U/mL	-	-
Bioreactor culture (final)	17.5	174.5 U/mL	100%	-
Culture after centrifugation	17	174 U/mL	96.9%	3.1%
Culture after microfiltration	17	174 U/mL	96.9%	3.1%
Culture after activation	17	225 U/mL	125%	-
Culture after ultrafiltration	3	1240 U/mL	121.8%	-
Sample after DEAE-Sepharose purification	3	990 U/mL	97.3%	2.7%
Sample after Q-Sepharose purification	0.291	4700 U/mL	44.8%	55.2%
Powder (after lyophilization with peptone casein)	38 g	36,000 U/g	44.8%	55.2%

**Table 2:** The milk-clotting activity of purified BacChym and BovChym tested on reconstituted cow’s, goat’s, ewes’, camel’s and mare’s milk.

Chymosin	Milk Type				
	Cow’s	Goat’s	Ewe’s	Camel’s	Mare’s
BacChym (U/mg)	16.590 ± 0.82	7850 ± 0.34	20.700 ± 0.85	13.140 ± 0.59	1966 ± 0.09
BovChym (U/mg)	12.854 ± 0.61	5385 ± 0.25	14.811 ± 0.72	10.013 ± 0.43	130.6 ± 5.5
Bovine chymosin from rennet (BioRen, Austria), U/mg	10.885 ± 0.42	6386 ± 0.37	8928 ± 0.35	ND *	ND *

* ND—not detected (no clotting).

**Table 3:** Indicators of cow’s, goat’s, ewes’, camel’s and mare’s milk.

Milk	Fat (%)	Proteins (%)	Total Protein (%)	Lactose (%)	Salts (%)	Solid (%)	Density, g/cm^3^
**Cow’s**	3.36 ± 0.09	3.00 ± 0.12	2.98 ± 0.05	4.47 ± 0.04	0.67 ± 0.02	11.49 ± 0.33	1.028 ± 0.002
**Goat’s**	3.62 ± 0.05	3.02 ± 0.1	2.99 ± 0.07	4.49 ± 0.07	0.68 ± 0.04	11.78 ± 0.32	1.028 ± 0.002
**Ewes’**	4.32 ± 0.04	4.30 ± 0.04	4.24 ± 0.07	6.35 ± 0.05	0.96 ± 0.04	11.54 ± 0.34	1.041 ± 0.002
**Camel’s**	4.08 ± 0.08	2.94 ± 0.07	2.86 ± 0.06	4.29 ± 0.07	0.16 ± 0.06	11.86 ± 0.42	1.027 ± 0.002
**Mare’s**	1.14 ± 0.03	2.92 ±0.09	2.94 ± 0.04	4.40 ± 0.04	0.66 ± 0.03	9.14 ± 0.22	1.029 ± 0.002

**Table 4:** A comparison of parameters of cheese production with the lyophilized BacChym from cow’s, goat’s, ewes’, camel’s and mare’s milk.

Milk	Volume (L)	Amount of Added Lyophilized Chymosin (mg)	Whey Amount (L)	Postpress Cheese Yield (g)	Cheese Yield (%)	Moisture (%)	Yield of Solids (g)
Cow’s	1	0.026	0.73	180	18.0	32.0	122.4
Goat’s	1	0.052	0.69	173	17.3	44.4	96.18
Ewes’	1	0.026	0.81	159	15.9	28.0	114.3
Camel’s	1	0.083	0.83	104	10.4	25.7	77.27
Mare’s	1	0.234	0.9	30	3	26.6	22.02

**Table 5:** Protein profile (g/L) of milk from different mammalian species.

Protein Fraction	Cow	Goat	Ewe	Camel	Mare
Total casein	24.8–31.9	23.3–46.3	41.8–52.7	22.1–26.0	9.40–13.56
**Caseins**					
α_s1_-Casein	8.0–10.8	0–13	15.4–22.1	4.9–5.7	2.4
α_s2_-Casein	2.8–3.4	2.3–11.6	6–8	2.1–2.5	0.2
β-Casein	9.8–12.0	0–29.6	15.6–39.6	14.4–16.9	10.66
κ-Casein	4.2–6.7	3.5–13.4	3.2–12.2	0.8–0.9	0.24
**Whey proteins**					
β-Lactoglobulin	3.42–5.76	1.5–5.0	6.5–8.5	Absent	2.55
α-Lactoalbumin	0.63–0.89	0.7–2.3	1–1.9	0.8–3.5	2.37

* Sources: Adapted from [12,15,37,38].

## Data Availability

All datasets used and/or analyzed during the current study are available from the corresponding author on reasonable request.

## Supplementary Materials

The following supporting information can be downloaded at: https://www.mdpi.com/article/10.3390/biology11111545/s1, File S1: Original image of Western blot analysis.
