# Supplementary figures and images for: Obtaining of Recombinant Camel Chymosin and Testing Its Milk-Clotting Activity on Cow’s, Goat’s, Ewes’, Camel’s and Mare’s Milk
Source: Biology (Basel). 2022 Oct 22;11(11):1545. doi: 10.3390/biology11111545 (PMC9687658; doi:10.3390/biology11111545)

**Supplementary File S1:** Original image of SDS-PAGE analysis.

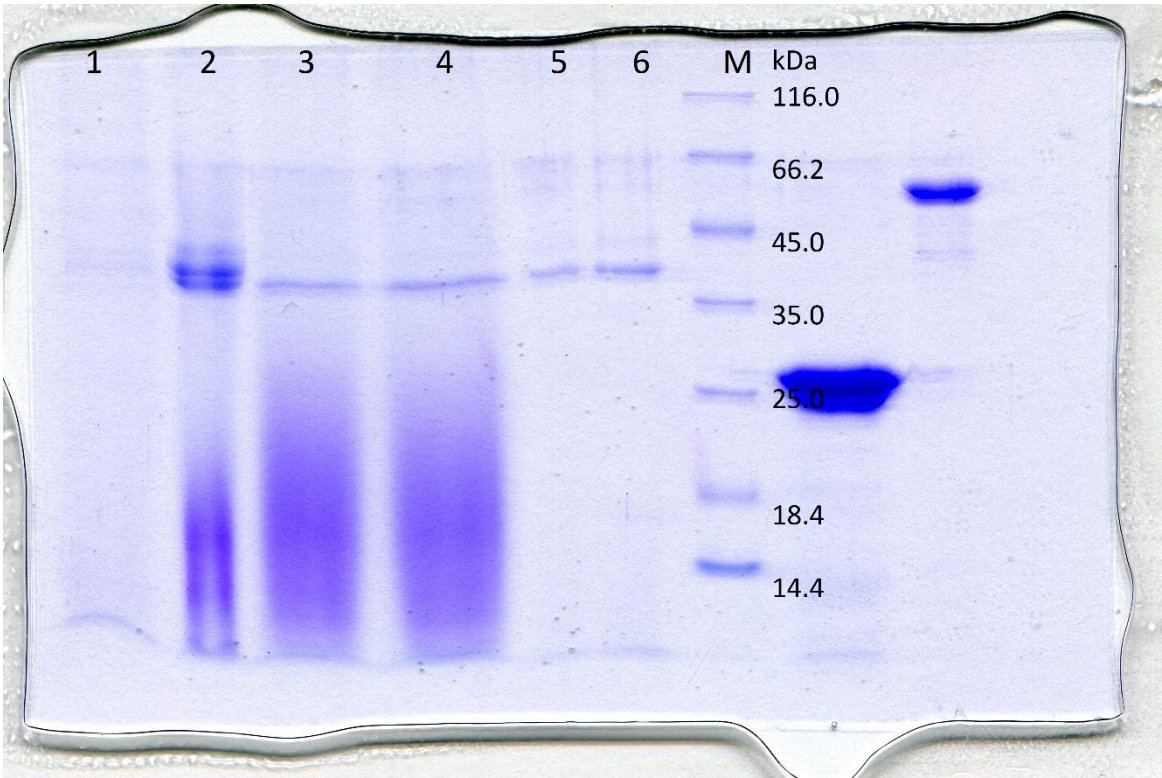

Supplement: Supplementary file 1 [file biology-11-01545-s001.zip › biology-1931770-supplementary.pdf]
